# Early Dynamics of Body Temperature in Acute Stroke: Insights into Outcomes and Management

**DOI:** 10.3390/jcm15124786

**Published:** 2026-06-19

**Authors:** Crhistian-Mario Oblitas, María Luz Alonso-Alonso, Antonio J. Mosqueira, Manuel Rodríguez-Yáñez, Iria López-Dequidt, Francisco Campos, Tomás Sobrino, José Castillo, Pablo Hervella, Ramón Iglesias-Rey

**Affiliations:** 1 Neuroimaging and Biotechnology Laboratory (NOBEL), Clinical Neurosciences Research Laboratory (LINC), Health Research Institute of Santiago de Compostela (IDIS), Hospital Clínico Universitario, Rúa Travesa da Choupana, s/n, 15706 Santiago de Compostela, Spain; 2 Neuroradiology Group, Health Research Institute of Santiago de Compostela (IDIS), 15706 Santiago de Compostela, Spain; 3 Stroke Unit, Department of Neurology, Hospital Clínico Universitario, 15706 Santiago de Compostela, Spain; 4 Stroke Unit, Department of Neurology, Hospital Clínico Universitario de A Coruña, 15006 A Coruña, Spain; 5 Translational Stroke Laboratory (TREAT), Clinical Neurosciences Research Laboratory (LINC), Health Research Institute of Santiago de Compostela (IDIS), 15706 Santiago de Compostela, Spain; 6 NeuroAging Laboratory (NEURAL), Health Research Institute of Santiago de Compostela (IDIS), 15706 Santiago de Compostela, Spain; 7 Centro de Investigación Biomédica en Red en Enfermedades Neurodegenerativas (CIBERNED), Instituto de Salud Carlos III, 28220 Madrid, Spain

**Keywords:** disability, hyperthermia, ischemic stroke, hemorrhagic stroke

## Abstract

**Background:** Following a stroke, body and brain temperatures are closely linked. Elevated temperature may reflect the severity of brain injury rather than infection. The significance of admission temperature remains unclear, and hypothermia treatment lacks proven efficacy and safety. Administering paracetamol (acetaminophen) above 36.5 °C is considered safe, though its clinical benefit is modest. This study aimed to examine how admission temperature, peak temperature in the first 24 h, and temperature fluctuations affect three-month functional outcomes. **Methods:** We conducted a retrospective study using data from a prospective stroke registry, including 5883 patients (4830 with ischemic stroke [IS] and 1053 with hemorrhagic stroke [HS]). Temperature at admission, maximum temperature within the first 24 h, and the temperature increase during the first day were assessed. Patients with a temperature ≥ 37.5 °C received 3 g of paracetamol per day until normothermia was achieved. **Results:** Baseline temperature was not associated with 3-month functional outcomes. In IS patients, an increasing temperature during the first 24 h was associated with a 10-fold higher risk of poor functional outcome (sensitivity 81%, specificity 64%); whereas in HS, the risk increased sevenfold (sensitivity 88%, specificity 53%). The most reliable predictor of therapeutic response was the temperature increase on the first day, with sensitivities of 89% and 83%, and specificities of 84% and 71%, for IS and HS, respectively. **Conclusions:** An increase in temperature during the first 24 h, rather than a single measurement, is the most reliable temperature-based biomarker for predicting poor functional outcomes and guiding the initiation of antihyperthermic treatment.

## 1. Introduction

The association between hyperthermia (or fever) and poor stroke prognosis has been recognized since antiquity. However, it was not until 1976 that the first clinical study formally demonstrating this relationship was published [1]. A prospective analysis followed in 1994 [2], with numerous subsequent studies confirming the association [3,4,5]. Currently, the link between hyperthermia and stroke outcomes is well established and incorporated into clinical practice guidelines [6,7,8].

The critical window during which hyperthermia has the most adverse effects is within the first 48 to 72 h after symptom onset, regardless of whether the cause is infectious [9,10]. Although admission temperature has been widely studied, its prognostic value remains debated and is often considered inconsistent. In contrast, the strongest evidence indicates that temperature readings taken between 24 and 48 h after stroke are better predictors of clinical outcomes [3,4,11,12]. Importantly, the impact of temperature fluctuations on prognosis in both acute ischemic and hemorrhagic strokes has not been thoroughly assessed.

During an acute stroke, body and brain temperatures are closely linked. Preclinical and some clinical studies have shown that brain temperature, especially in the ischemic area, often exceeds systemic temperature, creating a harmful environment for neurons [13,14]. This correlation reflects complex pathophysiological processes triggered by interrupted blood flow to the brain. Notably, increased temperature is not always caused by infection; it can also stem from damage to brain regions that regulate temperature [15]. Excitotoxicity, oxidative stress, and both local and systemic inflammatory responses can raise temperature by 1–2 °C [14,15,16,17,18]. These mechanisms suggest that dynamic temperature fluctuations, rather than fixed values, might be more sensitive indicators of brain injury progression. In this setting, recently, a meta-analysis [12] found that the presence of fever was significantly associated with neurological deterioration (pooled odds ratio [OR] 1.10; 95% CI: 1.05–1.15), large infarct size (pooled OR 2.94; 95% CI: 2.90–2.98), hemorrhagic transformation (pooled OR 1.63; 95%CI: 1.34–1.97), and mortality (pooled OR 1.31; 95% CI: 1.28–1.34) outcomes, among others, in critically ill patients with acute brain injury.

In contrast, induced hypothermia has been proposed as a potential neuroprotective strategy. While it has demonstrated benefits in animal models and in the management of cardiorespiratory arrest [19,20,21], its application in stroke has yielded inconsistent results across clinical trials. Moreover, it is associated with significant complications, including pneumonia, coagulopathy, and cardiac arrhythmias. As such, its routine use is not recommended outside of controlled clinical trials [22,23].

On the other hand, antipyretic treatment, primarily with paracetamol (acetaminophen) or metamizole, is a simple, safe, and guideline-recommended approach aimed at maintaining body temperature below 37.5 °C, even in the absence of infection [6,7]. Clinical studies have shown that early administration of paracetamol can lower body temperature by approximately 0.3–0.5 °C, a reduction associated with functional improvement in some patients [24]. Although the clinical benefits are modest, the favorable safety profile and ease of implementation have established antipyretic therapy as the standard of care in febrile stroke management. A secondary analysis of the PAIS study [25] showed that administering 6 g of paracetamol daily and initiating treatment within 12 h of symptom onset is most efficacious among patients who develop fever within the first 24 h, compared with those at the time of admission, supporting the idea of early dynamic evaluation of hyperthermia after stroke.

Given this background, the present study systematically evaluates admission, peak, and dynamic temperature increases across a large cohort of ischemic and hemorrhagic stroke patients, addressing an important gap in understanding the prognostic value of temperature variation. We hypothesize that greater temperature fluctuation during the first 24 h is a more significant prognostic biomarker and a better predictor for selecting patients for normothermia therapy than admission temperature or the highest temperature recorded in the first 24 h. The main goal of this study was to examine how admission temperature, peak temperature within the initial 24 h, and temperature fluctuations influence functional outcomes at three months. The secondary aim was to explore how different temperature-related biomarkers affect the effectiveness of antihyperthermic treatment.

## 2. Materials and Methods

### 2.1. Patient Selection

This is a retrospective study based on a prospective biobank (BICHUS) of patients with stroke admitted to the Stroke Unit at the Hospital Clínico Universitario de Santiago de Compostela (Spain) between January 2008 and December 2018. A total of 6632 patients were included in the biobank.

This research adheres to the Good Clinical Practice guidelines, as set out in the Declaration of Helsinki (1964) and its subsequent revision in Fortaleza (2013). Both the BICHUS biobank and this research have been approved by the Galician Ethics Committee (project identification code 2019/616; 1 September 2004). Informed consent was obtained from all patients (or their authorized representatives) included in the study, and participant anonymity was maintained.

Exclusion criteria were as follows: (1) patients who died within the first 24 h (n = 139); (2) those without recorded temperature data (n = 86); (3) those lacking follow-up at 3 months ± 15 days (n = 174); (4) those without diagnostic confirmation by neuroimaging (n = 103); (5) patients who underwent surgical intervention (n = 38); and (6) those admitted more than 24 h after symptom onset or awakening (n = 209). After applying these criteria, a total of 5883 patients were included in the analysis, 4830 with a diagnosis of IS and 1053 with HS.

While hyperthermia has been suggested as a potential risk factor for poor neurological prognosis, the underlying mechanisms and pathophysiology differ significantly from those of IS and HS. Additionally, the sample sizes for each group in our cohort are different. Thus, to minimize potential bias, temperature was analyzed separately for the IS and HS groups.

### 2.2. Temperature

Axillary temperature was taken using the ICOeco^®^ device(Peroxfarma, Barcelona, Spain), with the patient’s armpit cleaned and dried beforehand. The thermometer was positioned in the center of the opposite limb affected by stroke, making direct skin contact with the armpit. The arm was kept firmly against the chest to fully close the armpit for 5 min. A 0.5 °C (Celsius degrees) correction was applied to all auxiliary readings, as recommended by the manufacturer, to obtain the final temperature used in the present study. Alcohol disinfectant was applied before and after each measurement, following the manufacturer’s instructions. The procedure was performed by nursing staff in the Hospital Emergency Department upon admission and subsequently every 6 h in the Stroke Unit.

For the present study, we defined 4 different variables regarding temperature in order to capture potential dynamic fluctuation of temperature: (a) the temperature on admission; (b) the maximum temperature, defined as the highest temperature recorded within the first 24 h excluding the admission temperature; (c) the difference between the maximum temperature in the first 24 h and the temperature on admission; and (d) change in temperature during the first 24 h (excluding temperature on admission), dichotomized as an increase when the difference was > 0 °C (Celsius degrees) (yes = 1), or an equal-to-or-decrease when the difference was ≤ 0 °C (Celsius degrees) (no = 0). Additionally, hyperthermia was defined as an axillary temperature ≥ 37.5 °C.

Following the Stroke Unit protocol, all patients with a temperature of 37.5 °C or higher on admission or within the first 24 h were treated with 1 g of oral paracetamol or 2 g of intravenous metamizole every 8 h until normothermia was achieved. No patients received hypothermic treatment. Infection detection and management followed the Stroke Unit protocols, the guidelines of the Hospital’s Microbiology Service, and the recommendations of the Spanish Society of Neurology [26].

For patients who received antihyperthermic treatment, a therapeutic response was considered positive if the difference between the maximum temperature in the first 24 h and the temperature on admission was <0 °C; otherwise, it was considered negative.

### 2.3. Clinical Variables and Neuroimaging Studies

Neurological deficit was assessed using the National Institutes of Health Stroke Scale (NIHSS), and functional outcome was evaluated using the modified Rankin Scale (mRS). Stroke etiology classification was made using the TOAST (Trial of Org 10,172 in Acute Stroke Treatment) criteria [27]. The NIHSS was measured upon admission, every 6 h during the first 24 h, and while the patient was not clinically stable. To evaluate the change in neurological deficit over the first 24 h, we calculated the percentage difference between the NIHSS at admission and 24 h using the formula: (NIHSS at admission − NIHSS at 24 h)/NIHSS at admission × 100 [28]. The modified Rankin Scale was assessed at 3 months ± 15 days. A good prognosis (or outcome) was defined as a Rankin score ≤ 2, and a poor prognosis as a score > 2 [29].

Cerebral infarct volume was measured using a CT scan performed between days 4th and 7th after admission. For patients with intracerebral hemorrhage, hematoma volume was determined on the admission CT scan. The volume of perihematomal edema was calculated as the difference between the total lesion volume on a CT scan performed between days 4 and 7 and the initial hematoma volume. These measurements were performed by neuroradiologists, blinded to clinical data.

### 2.4. Statistical Analysis

Non-continuous variables were expressed as percentages. The Kolmogorov–Smirnov test was used to determine the normality of continuous variables. Continuous variables with a normal distribution were expressed as mean ± 1 standard deviation, and those without a normal distribution were expressed as median [25th percentile 75th percentile]. To compare a noncontinuous variable in two populations, we used the chi-square test; for continuous variables with a normal distribution, we used the Student *t* test (with Levene’s test used to assume equal variances), and the Mann–Whitney Wilcoxon test for variables without a normal distribution. To compare a continuous variable in more than two populations, we used the ANOVA test.

To compare two variables in a single population, we used the Spearman correlation coefficient for noncontinuous and continuous variables with a normal distribution, and the Pearson r correlation coefficient for continuous variables with a normal distribution. For the multivariate study, multiple logistic regression analyses were performed, adjusting for all variables that reached statistical significance in the bivariate studies. The clinical relevance of the temperature biomarkers was determined by sensitivity (the likelihood of a positive test in patients) and specificity (the likelihood of a negative test in healthy individuals). Statistical evaluation of the temperature markers for a previously defined cutoff point in the methods related to prognosis was determined by ROC analysis. The power of these curves was quantified using the overall model quality and the area under the ROC curve.

## 3. Results

### 3.1. Sample Description

Of the 4830 patients eligible for this study with a diagnosis of IS and 1053 with non-traumatic HS, 55.5% were male (mean age 69.5 ± 12.8 years), and 44.5% were female (mean age 75.4 ± 14.1 years). Among IS patients, 768 underwent intravenous thrombolysis, 101 received endovascular therapy (including intra-arterial thrombolysis or mechanical thrombectomy), and 67 received both intravenous and endovascular therapy. According to the TOAST classification, 1101 patients (22.8%) were classified as atherothrombotic, 1758 (36.4%) as cardioembolic, 420 (8.7%) as lacunar, 1493 (30.9%) as undetermined, and 58 (1.2%) as others. In intracerebral hemorrhages, 483 cases (45.9%) were hypertensive, 111 (10.5%) amyloid-related, 150 (14.2%) due to anticoagulants, and 310 (29.4%) of undetermined cause.

The mean temperature on admission was 36.7 ± 0.6 °C, while the mean for maximum temperature within the first 24 h was 36.2 ± 0.8 °C. The mean difference between the maximum temperature at 24 h and the temperature on admission was −0.5 ± 0.6 °C. A total of 21.9% of patients experienced an increase in temperature within the first 24 h. Hyperthermia was present in 9.8% of patients upon admission, and in 5.9% during the first 24 h. In total, 11.3% of patients received antihyperthermic treatment during the first 24 h, and infection was identified in 7.0% of patients within this period (49.0% respiratory, 41.9% urinary, and 9.1% other sites). The therapeutic response to antihyperthermic treatment was positive in 75.4% of treated patients.

**Figure 1:**
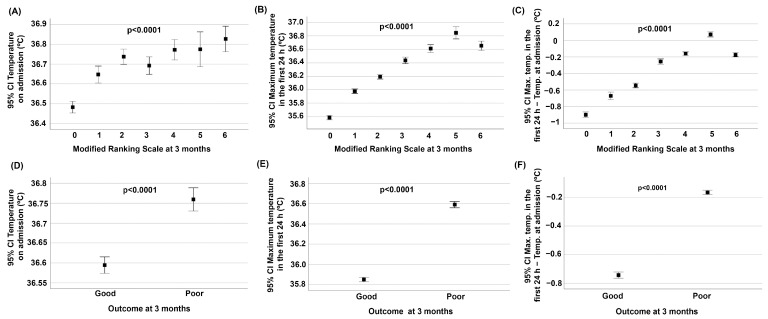
(**A**–**C**) Relationship between temperature with the modified Rankin Scale (**top**) and (**D**–**F**) with the 3-month functional outcome (**bottom**) in all patients included (ischemic and hemorrhagic strokes). The relationship is significant and progressive concerning the patients’ functional disability at 3 months.

The Spearman correlation coefficient between the mRS at 3 months and temperature on admission was 0.142 (*p* < 0.001), with the maximum temperature in the first 24 h being 0.562 (*p* < 0.001), and with the difference between the maximum temperature in the first 24 h and the temperature on admission being 0.688 (*p* < 0.001) (Figure 1).

Given that the temperature profiles of patients with ischemic and HS (Table 1) were different, statistical analysis was conducted separately for each diagnostic group rather than on the entire sample.

### 3.2. Temperature Biomarkers and Outcome in Patients with Ischemic Stroke

Variation in axillary temperature during the first 24 h was significantly associated with changes in neurological deficit over the same period (Supplementary Figure S1A). Neurological improvement was more pronounced in patients with a decrease in axillary temperature within the first 24 h.

Among patients with poor functional outcome at 3 months, both admission temperature and the maximum temperature in the first 24 h were significantly higher. Additionally, the increase in temperature within the first 24 h was greater in patients with poorer prognosis (Supplementary Table S1).

Given the relationship between the three temperature biomarkers and the 3-month outcome, we conducted different logistic regression models, incorporating each biomarker along with other variables identified as significant in the bivariate analysis (Supplementary Table S1). The logistic regression models revealed a non-significant odds ratio (OR) for temperature on admission (OR 1.15, 95% CI 0.94–1.40, *p* = 0.171). In contrast, significant associations were found for the maximum temperature in the first 24 h (OR 4.68, 95% CI 3.76–5.83, *p* < 0.001) and even more for the difference between the maximum temperature in the first 24 h and the temperature on admission (OR 8.24, 95% CI 6.28–10.83, *p* < 0.001). Finally, the increase in temperature during the first 24 h was associated with a 10-fold increase in the risk of poor outcome at 3 months, with a sensitivity of 81% and specificity of 64% (Table 2).

### 3.3. Temperature Biomarkers and Outcome in Patients with Hemorrhagic Stroke

Hematoma volume at admission showed a modest correlation with temperature on admission (Pearson’s coefficient = 0.164, 95% CI 0.100–0.227, *p* < 0.001). However, this relationship was stronger when examining the difference in temperature over the first 24 h and perihematomal edema. Higher temperature was associated with greater edema volume (Supplementary Figure S1B).

Temperature on admission (36.7 ± 0.6 vs. 36.8 ± 0.8 °C) and the maximum temperature within the first 24 h (36.0 ± 0.7 vs. 36.7 ± 0.8 °C) were significantly higher in patients with poor outcomes. Furthermore, the difference between the maximum temperature in the first 24 h and the temperature on admission was more pronounced in patients with a poor outcome (−0.7 ± 0.6 vs. −0.1 ± 0.4 °C) (Supplemental Table S2).

In the logistic regression models (Table 3), only the maximum temperature in the first 24 h and, notably, the difference between the maximum temperature in the first 24 h and the temperature on admission remained significantly associated with the 3-month outcome after adjustment for the variables identified as significant in the bivariate analysis (Supplementary Table S2). The increase in temperature during the first 24 h was associated with a 17-fold higher risk of poor outcome at 3 months, with a sensitivity of 88% and a specificity of 53%.

### 3.4. Efficacy of the Response to Antihyperthermic Treatment According to the Therapeutic Biomarker: Ischemic Stroke

Of the 4830 patients with IS, 523 received antihyperthermic treatment; 78.6% demonstrated a positive therapeutic response. Among those who responded positively, 70.2% had a poor prognosis at 3 months. There was no significant difference in their admission temperatures (37.9 ± 0.3 vs. 37.9 ± 0.4 °C; *p* = 0.907). However, the maximum temperature recorded within the first 24 h was significantly higher in patients with poor outcomes (36.7 ± 0.5 vs. 37.4 ± 0.4 °C; *p* < 0.001). Concordantly, patients with poor outcomes showed a significantly smaller difference between the maximum temperature in the first 24 h and the temperature on admission (−0.5 ± 0.4 °C vs. −1.2 ± 0.5 °C, *p* < 0.001).

**Figure 2:**
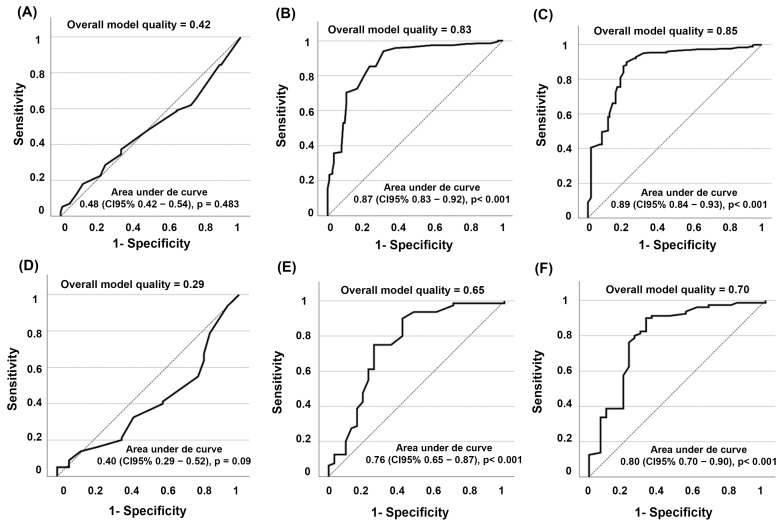
ROC curves for predicting poor 3-month functional outcome in patients with acute stroke (baseline temperature ≥ 37.5 °C) following antihyperthermic treatment. Panels (**A**–**C**) represent patients with IS, using the following temperature markers as predictors: (**A**) Temperature at admission, (**B**) Maximum temperature within the first 24 h, (**C**) Any increase in temperature over the first 24 h (≥0 °C). Panels (**D**–**F**) show the same analysis in patients with hemorrhagic stroke, using: (**D**) Temperature at admission, (**E**) Maximum temperature within the first 24 h, (**F**) A temperature increase of ≥0 °C during the first 24 h.

A cutoff temperature ≥ 37.5 °C on admission did not reliably identify patients at risk of poor 3-month outcomes (sensitivity 62%, specificity 36%). However, a maximum temperature ≥ 37.5 °C within the first 24 h was a significant predictor of poor outcomes, with a sensitivity of 81% and specificity of 80%. Furthermore, a temperature difference ≥ 0 °C between the maximum temperature in the first 24 h and the temperature on admission was highly predictive of poor outcomes, with a sensitivity of 89% and a specificity of 84% (Figure 2A).

### 3.5. Efficacy of the Response to Antihyperthermic Treatment According to the Therapeutic Biomarker: Hemorrhagic Stroke

Of the 1053 patients with HS, 109 received antihyperthermic treatment, 66.1% showed a positive therapeutic response. Among these patients, 27.9% had a good outcome at 3 months, while 72.1% had a poor outcome. There was no significant difference in their admission temperatures (38.0 ± 0.3 vs. 37.9 ± 0.4 °C; *p* = 0.467). However, the maximum temperature recorded within the first 24 h was significantly higher in those with a poor outcome (36.9 ± 0.6 vs. 37.4 ± 0.5 °C, *p* < 0.001). Concordantly, patients with poor outcomes showed a significantly smaller difference between the maximum temperature in the first 24 h and the temperature on admission (−0.6 ± 0.5 °C vs. −1.1 ± 0.5, *p* < 0.001).

A cutoff temperature of ≥37.5 °C upon admission in patients receiving antihyperthermic treatment did not reliably predict those at risk of a poor outcome at 3 months (sensitivity 41%, specificity 42%). A maximum temperature ≥ 37.5 °C during the first 24 h had lower sensitivity (55%) but higher specificity (68%). However, the strongest predictive value was found in the difference between the maximum temperature in the first 24 h and the temperature on admission, with a cutoff of ≥0 °C, which exhibited a sensitivity of 83% and a specificity of 71% (Figure 2B).

## 4. Discussion

Hyperthermia within the first 72 h after symptom onset, even when moderate (≥37.5 °C), is one of the most significant predictors of outcome in patients with both ischemic and hemorrhagic strokes. However, the relationship between baseline temperature and outcome remains uncertain, and our study confirms the absence of such an association. The presence of infection during this period (6.2% in patients with IS and 10.2% in patients with intracerebral hemorrhage;) may be considered a pre-existing condition unrelated to the stroke outcome (*p* = 0.166;).

**Figure 3:**
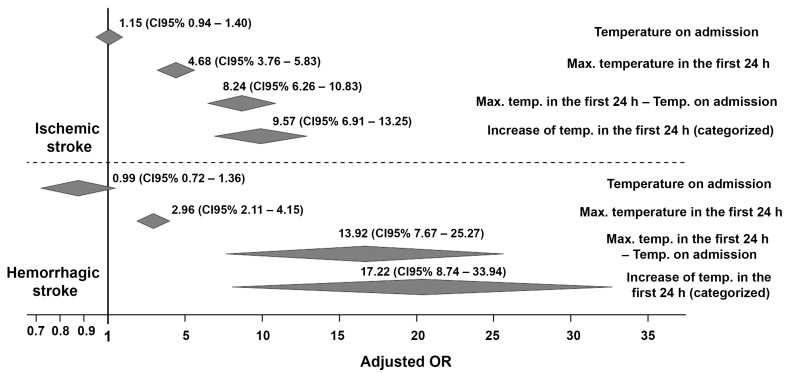
Adjusted odds ratio (OR) for the dependent variable of poor functional outcome at 3 months in patients with IS and HS, according to the different temperature biomarkers used. Temperature increase was categorized as 1 = increased temperature in the first 24 h, 0 = equal or lower temperature relative to baseline.

While the maximum temperature in the first 24 h is a significant prognostic marker, our findings indicate that the increase in temperature, rather than a specific threshold at a given time, is the strongest predictor of outcome. These results are consistent across both ischemic and HS (Figure 3). This rise in systemic temperature appears to be independent of other systemic factors. Experimental studies have shown that hyperthermia not only results from increased brain injury but can also exacerbate it through mechanisms such as excitotoxicity, oxidative stress, and inflammation [15,16,19,30,31]. Hyperthermia due to the involvement of brain thermal-regulation centers is relatively rare.

Clinically, an association between brain injury markers and hyperthermia has also been observed [32,33,34,35,36]. In a sample of 580 patients with IS, for whom Diffusion-Weighted Imaging (DWI) was available at admission, we confirmed that the difference between infarct volume measured on a CT scan (performed between days 4 and 7) and the lesion volume at admission (determined by DWI) showed a strong and direct relationship with temperature increase (Pearson’s r coefficient = 0.714, *p* < 0.001; in the results). Furthermore, we have also observed a relationship between increased temperature and perihematomal edema. These findings suggest that higher temperatures may serve as a useful predictor of poor patient outcomes. Specifically, 82.1% of stroke patients who experienced temperature increases within the first 24 h developed poor outcomes at 3 months, compared to only 38.0% of those who did not experience any temperature change, regardless of the change’s magnitude. An isolated temperature measurement, even when elevated, has lower predictive value than changes in temperature over time.

The importance of identifying a sensitive and specific biomarker extends beyond its diagnostic and prognostic value, particularly in its therapeutic utility. Attempts to use hypothermic therapy to induce body temperatures between 32 and 34 °C have demonstrated neuroprotective effects. However, these efforts have been limited by a high incidence of complications, including fatal ones, which have confined their use to controlled clinical trials. Additionally, the complexity of managing hypothermia poses significant challenges, making it less feasible for widespread clinical use, especially for many patients [37,38,39,40,41].

Given the limitations of hypothermic therapy at temperatures between 32 and 34 °C, one study suggested that achieving a target temperature of 36 °C might be just as effective while being significantly safer [42]. In patients with acute stroke, treatment with high doses of paracetamol (6 g/day) has been shown to reduce body temperature by approximately 0.3 °C within 4 h of starting treatment [43]. This evidence supported the PAIS trials, where 6 g/day of paracetamol was administered to all patients with a body temperature ≥ 36.5 °C upon admission. Although the results were inconclusive, a modest 5% reduction in the absolute risk of poor prognosis was observed, with no at-risk serious adverse events in the paracetamol arm [23,43]. However, a subsequent study found that preventive administration of paracetamol to all patients with acute stroke offers no benefit [44].

In this context, our study uniquely examined how different temperature metrics influence prognosis and guide responses to antihyperthermic therapy. Unlike previous trials that used preventive treatment or treated patients with temperatures ≥ 36.5 °C, we administered paracetamol (3 g/day) or metamizole (2 g every 8 h) only to those with temperatures ≥ 37.5 °C until they reached normothermia. Our findings indicate that overall hyperthermia (≥37.5 °C) is linked to poor 3-month outcomes. Nevertheless, subsequent analysis revealed that admission temperature alone exhibited limited predictive capacity in both IS and HS cohorts. This finding underscores that a single temperature measurement upon admission possesses minimal prognostic value and highlights the need for repeated temperature assessments during the initial phase characterized by dynamic fluctuations. Such an approach renders it an inadequate trigger for antihyperthermic therapy, except when the temperature exceeds 38.5 °C, as recommended by certain guidelines. Conversely, the onset of hyperthermia within the first 24 h, particularly the magnitude of the temperature elevation during this period, was significantly correlated with poorer functional outcomes at three months. Additionally, it was observed that reliance on baseline temperature to direct treatment had no discernible impact on outcomes. Initiating therapy in patients whose temperature increased during the first day proved markedly more effective, achieving both high sensitivity and specificity. Consequently, administering antihyperthermic treatment to acute stroke patients with an axillary temperature below 37.5 °C appears to be ineffective. Conversely, administering 3 g/day of paracetamol to those who exhibited an increase in temperature within the first 24 h was associated with an improved 3-month prognosis. Importantly, this therapeutic benefit was consistent across both ischemic and hemorrhagic stroke subtypes. Further well-designed randomized clinical trials are needed to confirm or refute this finding.

Our study has several limitations. First, it is a retrospective analysis rather than a prospective study, relying on data from a prospective registry not specifically designed to evaluate temperature metrics. Second, the registry captured a broad array of clinical, neuroimaging, and laboratory variables primarily to monitor the overall quality of care. Consequently, although our findings suggest a possible link between increased temperature and greater brain injury, the design lacks mechanistic insights into temperature dynamics and does not assess whether continuous or less frequent temperature measurements could provide better information about temperature fluctuations, or whether different locations, such as tympanic temperature, would yield better information. Third, complex powered individual longitudinal statistical analyses for dynamic variable changes in the temperature variable could not be performed. Instead, dynamic changes were estimated indirectly by combining different values and categorizing them within inherent limitations. Fourth, although the broad infectious source of fever was taken into account in our analysis, data on the severity and spectrum of antimicrobial therapies were not recorded, which may introduce potential bias. Fifth, the exclusion of small-group patients who died within the first 24 h may be a limitation for generalizing our results, as their more severe strokes may have influenced temperature dynamics and neuroimaging changes, which could not be fully assessed in this subgroup. Nonetheless, our analysis shows several strengths, including access to a large real-life cohort of stroke patients, all managed by specialized, stroke-trained teams in a dedicated unit, following standardized protocols. This setting enhances the generalizability and reliability of our observations.

## 5. Conclusions

In summary, monitoring temperature changes, specifically the rise in body temperature during the first 24 h after stroke onset, provides the most robust indicator for initiating antihyperthermic therapy. Targeting patients who exhibit a temperature increase over this period, rather than relying on a single admission measurement, may optimize treatment efficacy and improve functional outcomes. A randomized clinical trial is warranted to validate these retrospective findings prior to establishing evidence-based guidelines.

## Figures and Tables

**Table 1:** Temperature profile in patients with ischemic and hemorrhagic strokes.

	Ischemic Stroke n = 4830	Hemorrhagic Stroke n = 1053	*p*
Temperature on admission, °C	36.7 ± 0.6	36.8 ± 0.7	<0.001
Maximum temperature in the first 24 h, °C	36.2 ± 0.7	36.4 ± 0.8	<0.001
Difference between maximum temperature in the first 24 h and temperature on admission, °C	−0.5 ± 0.6	−0.4 ± 0.5	<0.001

**Table 2:** Logistic regression models in ischemic stroke. Dependent variable: 3-month outcome.

**Model A.**						
	**Not Adjusted**			Adjusted *		
	**OR**	**CI 95%**	***p***	**OR**	**CI 95%**	***p***
Temperature on admission	1.52	1.39–1.67	<0.001	1.15	0.94–1.40	0.171
**Model B.**						
	**Not Adjusted**			Adjusted *		
	**OR**	**CI 95%**	***p***	**OR**	**CI 95%**	***p***
Maximum temperature in the first 24 h	6.02	5.34–6.78	<0.001	4.68	3.76–5.83	<0.001
**Model C.**						
	**Not Adjusted**			Adjusted *		
	**OR**	**CI 95%**	***p***	**OR**	**CI 95%**	***p***
Difference between maximum temperature in the first 24 h and temperature on admission, °C	11.92	10.18–13.94	<0.001	8.24	6.26–10.83	<0.001
**Model D.**						
	**Not Adjusted**			Adjusted *		
	**OR**	**CI 95%**	***p***	**OR**	**CI 95%**	***p***
Increase in temperature in the first 24 h (categorized)	7.50	6.34–8.88	<0.001	9.57	6.91–13.25	<0.001

* Adjusted by: Time of onset of symptoms–hospital, women, age, high blood pressure, diabetes, smoker, alcoholism, atrial fibrillation, blood glucose, leukocytes, fibrinogen, C-reactive protein, reperfusion therapy, lesion volume on CT 4th–7th days, NIHSS on admission.

**Table 3:** Logistic regression models in hemorrhagic stroke. Dependent variable: 3-month outcome.

**Model A.**						
	**Not Adjusted**			Adjusted *		
	**OR**	**CI 95%**	***p***	**OR**	**CI 95%**	***p***
Temperature on admission	1.31	1.11–1.59	0.002	0.99	0.72–1.36	0.961
**Model B.**						
	**Not Adjusted**			Adjusted *		
	**OR**	**CI 95%**	***p***	**OR**	**CI 95%**	***p***
Maximum temperature in the first 24 h	3.95	3.18–4.92	<0.001	2.96	2.11–4.15	<0.001
**Model C.**						
	**Not Adjusted**			Adjusted *		
	**OR**	**CI 95%**	***p***	**OR**	**CI 95%**	***p***
Difference between maximum temperature in the first 24 h and temperature on admission, °C	16.93	11.63–24.66	<0.001	13.92	7.67–25.27	<0.001
**Model D.**						
	**Not Adjusted**			Adjusted *		
	**OR**	**CI 95%**	***p***	**OR**	**CI 95%**	***p***
Increase in temperature in the first 24 h (categorized)	8.00	5.30–12.09	<0.001	17.22	8.74–33.94	<0.001

* Adjusted by: Age, smoker, atrial fibrillation, blood glucose, leukocytes, fibrinogen, C-reactive protein, hematoma volume on CT at admission, NIHSS on admission.

## Data Availability

The statistical analysis plan is available on request. The data bank is not available for legal and ethical reasons.

## Supplementary Materials

The following supporting information can be downloaded at: https://www.mdpi.com/article/10.3390/jcm15124786/s1, Figure S1: (A) Correlation between temperature difference during the first 24 h and percentual variation in neurological deficit within the first 24 h (NIHSS at admission – NIHSS at 24 h/NIHSS at admission × 100), in patients with ischemic stroke. (B) Correlation between temperature difference within the first 24 h and volume of perihematomal edema; Table S1: Bivariate analysis among the ischemic stroke cohort. Dependent variable: Outcome at 3 months after ischemic stroke; Table S2: Bivariate analysis among the intracerebral hemorrhage cohort. Dependent variable: Outcome at 3 months after ischemic stroke.
