# Supplementary material for: Early Dynamics of Body Temperature in Acute Stroke: Insights into Outcomes and Management
Source: J Clin Med. 2026 Jun 19;15(12):4786. doi: 10.3390/jcm15124786 (PMC13302722; doi:10.3390/jcm15124786)

Supplemental material

Table S1. Bivariate analysis among the ischemic stroke cohort. Dependent variable: Outcome at 3 months after ischemic stroke.

	**Good**<br>**n = 2,539**	**Poor**<br>**n = 2,291**	**p**
Time of onset of symptoms – hospital, min	226.3 ± 166.1	257.5 ± 167.4	< 0.001
Women, %	39.3	51.0	< 0.001
Age, years	68.9 ± 13.3	75.6 ± 13.1	< 0.001
High blood pressure, %	61.7	66.0	< 0.001
Diabetes, %	23.3	25.1	0.082
Smoker, %	19.6	12.8	< 0.001
Alcoholism, %	10.5	12.8	0.007
Atrial fibrillation, %	14.4	29.4	< 0.001
Blood glucose, mg/dL	132.0 ± 54.1	145.4 ± 63.1	< 0.001
Leukocytes x 10^3/mL	8.6 ± 2.9	9.9 ± 3.4	< 0.001
Fibrinogen, mg/dL	427.8 ± 100.1	465.5 ± 102.1	< 0.001
C-reactive protein, mg/L	2.7 ± 3.5	4.9 ± 4.6	< 0.001
Reperfusion therapy, %	33.1	17.3	< 0.001
Lesion volume on CT 4th-7th days, mL	17.1 ± 32.8	90.8 ± 98.9	< 0.001
NIHSS on admission	11 [6, 16]	17 [12, 22]	< 0.001
Temperature on admission, °C	36.6 ± 0.5	36.8 ± 0.7	< 0.001
Maximum temperature in the first 24 h, °C	35.9 ± 0.6	36.6 ± 0.7	< 0.001
Maximum temperature in the first 24 h – Temperature on admission, °C	- 0.7 ± 0.6	- 0.2 ± 0.4	< 0.001

Table S2. Bivariate analysis among the intracerebral hemorrhage cohort. Dependent variable: Outcome at 3 months after ischemic stroke.

	**Good**<br>**n = 464**	**Poor**<br>**n = 636**	**p**
Time of onset of symptoms – hospital, min	237.2 ± 209.2	235.9 ± 216.7	0.761
Women, %	41.4	45.9	0.242
Age, years	72.0 ± 12.0	76.4 ± 11.7	< 0.001
High blood pressure, %	59.3	61.8	0.216
Diabetes, %	18.1	22.0	0.129
Smoker, %	12.7	9.3	0.043
Alcoholism, %	15.3	15.4	0.516
Atrial fibrillation, %	13.4	21.5	< 0.001
Blood glucose, mg/dL	128.3 ± 42.2	143.0 ± 46.8	< 0.001
Leukocytes x 10^3/mL	7.9 ± 2.5	8.8 ± 3.4	< 0.001
Fibrinogen, mg/dL	421.8 ± 89.2	456.2 ± 106.3	< 0.001
C-reactive protein, mg/L	3.5 ± 3.5	5.8 ± 5.5	< 0.001
Hematoma volume on CT at admission, mL	20.4 ± 18.5	50.7 ± 56.7	< 0.001
NIHSS on admission	9 [4, 13]	16 [11, 20]	< 0.001
Temperature on admission, °C	36.7 ± 0.6	36.8 ± 0.8	< 0.001
Maximum temperature in the first 24 h, °C	36.0 ± 0.7	36.7 ± 0.8	< 0.001
Maximum temperature in the first 24 h – Temperature on admission, °C	- 0.7 ± 0.6	- 0.1 ± 0.4	< 0.001

Figure

Figure S1: (A) Correlation between temperature difference during the first 24 h and percentual variation in neurological deficit within the first 24 h (NIHSS at admission – NIHSS at 24 h / NIHSS at admission x 100), in patients with ischemic stroke. **(B)** Correlation between temperature difference within the first 24 h and volume of perihematomal edema.

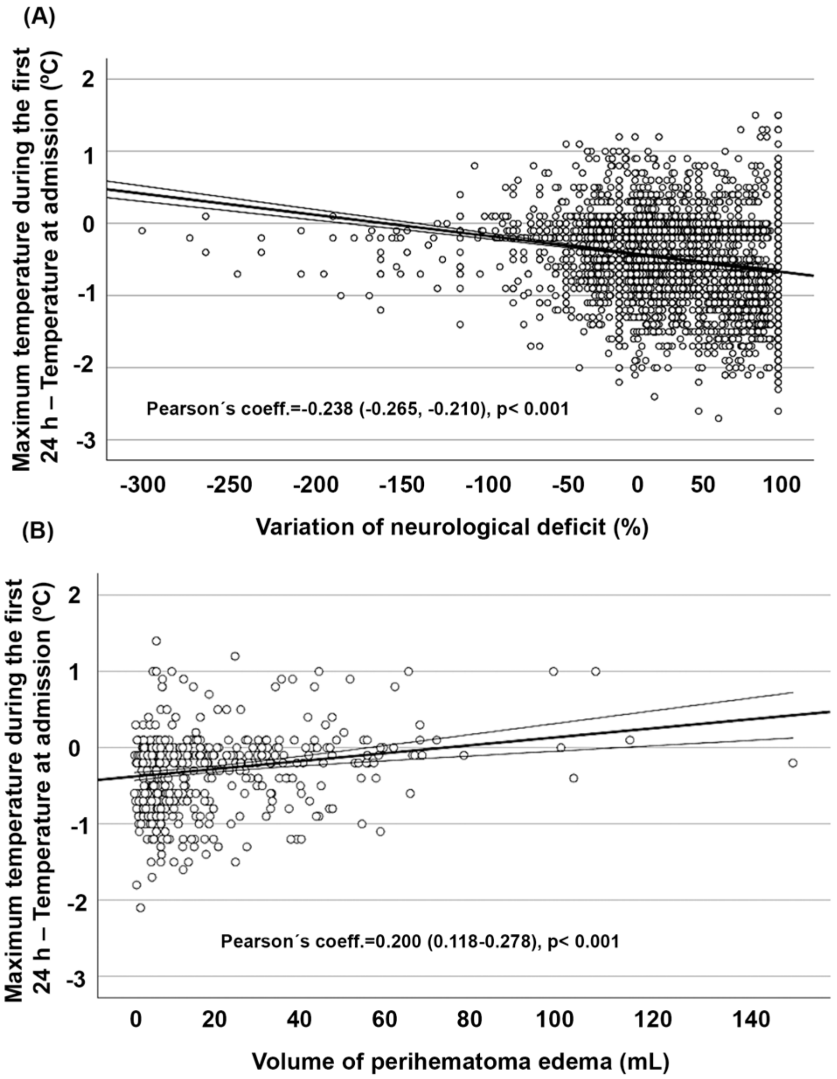

Supplement: Supplementary file 1 [file jcm-15-04786-s001.zip › jcm-4348788-supplementary.pdf]
